# Complete Genome Sequence of Stenotrophomonas maltophilia Podophage Paxi

**DOI:** 10.1128/mra.00179-22

**Published:** 2022-04-04

**Authors:** Eunhye Jeon, Alexis Hudson, Andrew Talcott, James Clark, Tram Le, Ben Burrowes, Mei Liu

**Affiliations:** a Department of Biochemistry and Biophysics, Texas A&M University, College Station, Texas, USA; b Center for Phage Technology, Texas A&M University, College Station, Texas, USA; c BB Phage Consultancy, LLC, Georgetown, Texas, USA; Loyola University Chicago

## Abstract

Stenotrophomonas maltophilia is a multidrug-resistant nosocomial pathogen that can cause life-threatening infections among immunocompromised populations. This report presents the complete 74,962-bp genome of S. maltophilia podophage Paxi, an N4-like phage sharing 85.3% nucleotide similarity to S. maltophilia podophage Pokken.

## ANNOUNCEMENT

Stenotrophomonas maltophilia is an environmentally ubiquitous and commensal bacterium, and it has also emerged as a nosocomial pathogen capable of causing life-threatening infections, especially among immunocompromised individuals (1). Treatment of this pathogen can be difficult as some strains are multidrug resistant (1, 2). To explore alternative strategies for controlling this pathogen, we report here the isolation and genome sequencing of S. maltophilia phage Paxi.

Phage Paxi was isolated from a pond water sample collected in September 2019 in Madisonville, TX (global positioning system [GPS] coordinates, 30.972534, −95.846840), using S. maltophilia ATCC 17807 as the propagation host. The host was aerobically cultured in tryptone nutrient broth or agar (0.5% tryptone, 0.25% yeast extract, 0.1% glucose, 0.85% NaCl, wt/vol) at 30°C, and phage propagation was performed using the soft agar overlay method (3). The genomic DNA of Paxi was purified using a modified Promega Wizard DNA cleanup kit protocol as previously described (4). DNA libraries were prepared as 300-bp inserts using a Swift 2S Turbo kit and sequenced on an Illumina MiSeq instrument with paired-end 150-bp reads using v2 300-cycle chemistry. A total of 159,286 raw reads were quality controlled using FastQC (www.bioinformatics.babraham.ac.uk/projects/fastqc) and FASTX-Toolkit v0.0.14 (http://hannonlab.cshl.edu/fastx_toolkit/) to yield 84,373 trimmed reads, from which a contig was assembled with 80-fold coverage using SPAdes v3.5.0 (5). Closure of the contig ends was accomplished by Sanger sequencing the PCR product, amplifying the end regions using the primers 5′-ATGGAGCCGGAGAGATCCTT-3′ (forward) and 5′-ACTTCATCAAGCGTGTCGGT-3′ (reverse). The CPT Galaxy-Apollo phage annotation platform (https://cpt.tamu.edu/galaxy-pub) was utilized for genome annotation (6–8). Structural annotation was performed using Glimmer v3 and MetaGeneAnnotator v1.0, and tRNA genes were detected using ARAGORN v2.36 and tRNAScan-SE v2.0 (9–12). Gene function was predicted using InterProScan v5.48, BLAST v2.9.0 against the NCBI nonredundant and UniProtKB Swiss-Prot databases, TMHMM v2.0, HHpred, and LipoP v1.0 (13–18). The genome-wide DNA sequence similarity to other phages was calculated using ProgressiveMauve v2.4 (19). All tools were run with default settings.

**FIG 1:**
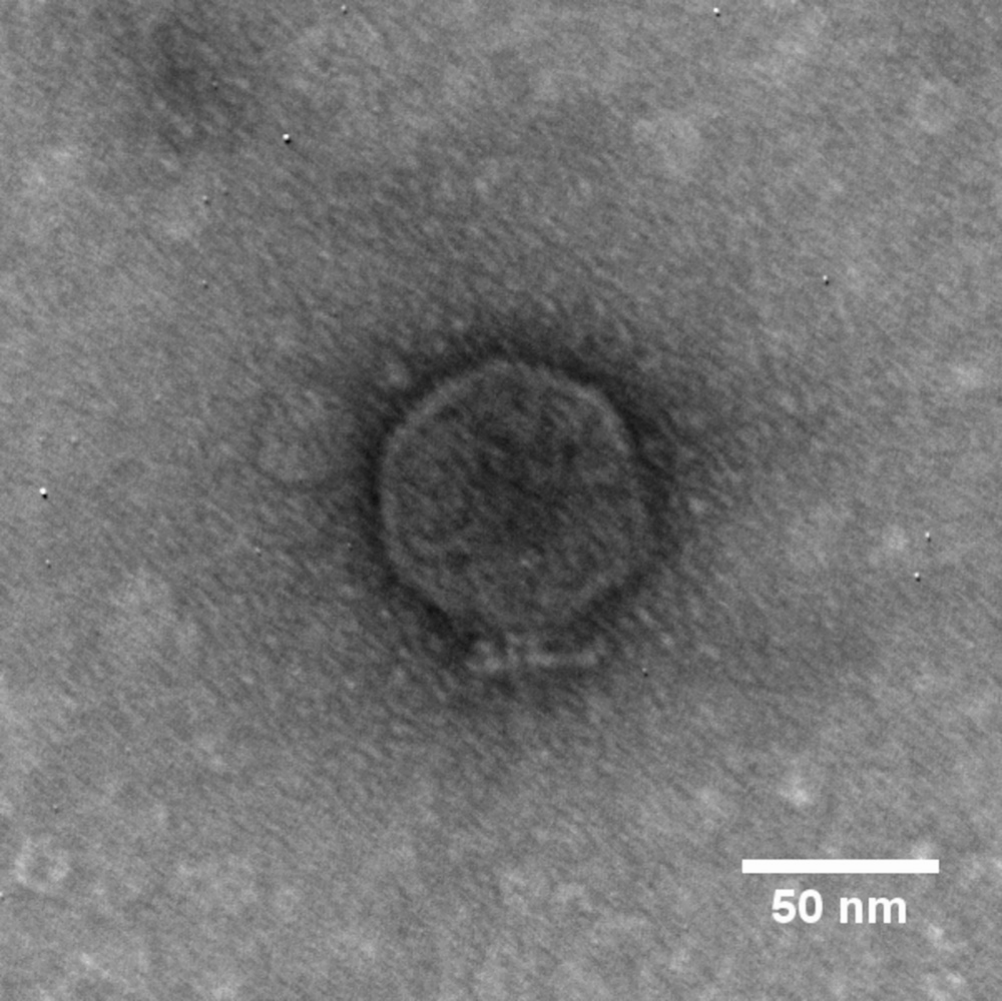
Transmission electron micrograph (TEM) of phage Paxi. Phage particles were diluted with TEM buffer (20 mM NaCl, 10 mM Tris-HCl [pH 7.5], 2 mM MgSO_4_) and captured on a freshly glow-discharged, Formvar carbon-coated grid. The grids were stained with 2% (wt/vol) uranyl acetate and observed on a Jeol 1200 EX TEM at 100 kV accelerating voltage at the Microscopy and Imaging Center at Texas A&M University.

Phage Paxi was determined to have a podovirus-like morphology (Fig. 1) by viewing samples negatively stained with 2% (wt/vol) uranyl acetate via transmission electron microscopy at the Texas A&M Microscopy and Imaging Center. Paxi has a complete genome sequence of 74,962 bp with a GC content of 54.6%, which is lower than its host’s average of 66.4% (2). A total of 89 protein-coding genes and 5 tRNA genes were predicted, yielding a coding density of 92.0%. A total of 25 protein-coding genes were assigned putative functions, including a lysis cassette consisting of a class II holin, a SAR endolysin, and a two-component spanin with embedded gene architecture. Genome-wide DNA sequence similarity based on ProgressiveMauve revealed that Paxi is 85.3% similar to *Stenotrophomonas* phage Pokken (GenBank accession number NC_049463.1) (20), and BLASTp (E value, <0.001) showed that 81 out of 89 proteins of Paxi are similar to those of Pokken. Like Pokken, Paxi demonstrates similarity to Enterobacteria phage N4 (NC_008720.1), sharing 43 similar proteins (BLASTp; E value, <0.001) such as virion RNA polymerase (NCBI protein accession number YP_950528.1) and an SAR endolysin N-acetylmuramidase (YP_950539.1). Paxi was predicted by PhageTerm to contain 538-bp direct terminal repeats.

### Data availability.

The genome sequence for Paxi was deposited in GenBank under accession number MZ326856. The associated BioProject, SRA, and BioSample accession numbers are PRJNA222858, SRR14095256, and SAMN18509291, respectively.
